# Efficient Neutrophil Extracellular Trap Induction Requires Mobilization of Both Intracellular and Extracellular Calcium Pools and Is Modulated by Cyclosporine A

**DOI:** 10.1371/journal.pone.0097088

**Published:** 2014-05-12

**Authors:** Anurag Kumar Gupta, Stavros Giaglis, Paul Hasler, Sinuhe Hahn

**Affiliations:** 1 Laboratory for Prenatal Medicine, Department of Biomedicine, University Hospital Basel, Basel, Switzerland; 2 Department of Rheumatology, Cantonal Hospital Aarau, Aarau, Switzerland; The Hospital for Sick Children and The University of Toronto, Canada

## Abstract

Excessive or aberrant generation of neutrophil extracellular traps (NETs) has recently become implicated in the underlying aetiology of a number of human pathologies including preeclampsia, systemic lupus erythromatosus, rheumatoid arthritis, auto-antibody induced small vessel vasculitis, coagulopathies such as deep vein thrombosis or pulmonary complications. These results imply that effective pharmacological therapeutic strategies will need to be developed to counter overt NETosis in these and other inflammatory disorders. As calcium flux is implicated in the generation of reactive oxygen species and histone citrullination, two key events in NETosis, we analysed the roles of both extra- and intracellular calcium pools and their modulation by pharmacological agents in the NETotic process in detail. Interleukin-8 (IL-8) was used as a physiological stimulus of NETosis. Our data demonstrate that efficient induction of NETosis requires mobilisation of both extracellular and intracellular calcium pools. Since modulation of the calcineurin pathway by cyclosporine A has been described in neutrophils, we investigated its influence on NETosis. Our data indicate that IL-8 induced NETosis is reduced by ascomycin and cyclosporine A, antagonists of the calcineurin pathway, but not following treatment with rapamycin, which utilizes the mTOR pathway. The action of the G protein coupled receptor phospholipase C pathway appears to be essential for the induction of NETs by IL-8, as NETosis was diminished by treatment with either pertussis toxin, a G-protein inhibitor, the phospholipase C inhibitor, U73122, or staurosporine, an inhibitor of protein kinase C. The data regarding the calcineurin antagonists, ascomycin and cyclosporine A, open the possibility to therapeutically supress or modulate NETosis. They also provide new insight into the mechanism whereby such immune suppressive drugs render transplant patients susceptible to opportunistic fungal infections.

## Introduction

Neutrophil extracellular traps (NETs), generated by a process termed NETosis, are a novel mechanism used by the innate immune system to ensnare and kill invading pathogens [1], [2]. NETs are generated in response to a number of pathological, physiological and pharmacological stimuli [3]. These include microorganisms, inflammatory cytokines, pharmacological agents (phorbol esters or calcium ionophores), IL-8 associated with placental micro-particles or anti-neutrophil cytoplasmic antibodies (ANCA) [1]–[6]. NETs consist of a histone rich DNA backbone decorated with granular proteins, which have been suggested to contribute to their anti-microbial action, but may also play a role in NETosis-associated tissue damage, particularly of endothelial or alveolar cells [3], [5], [7]–[9].

In the latter context a number of studies have indicated that aberrant NETosis may play a role in the underlying aetiology of a number of inflammatory human pathologies including preeclampsia, systemic lupus erythromatosus, rheumatoid arthritis, auto-antibody induced small vessel vasculitis and psoriasis [3], [4], [9]–[12]. In addition, NETs have become implicated in thrombosis, particularly deep vein thrombosis, by providing a scaffold for the coagulation process [13], [14]. Furthermore, NETosis may contribute to alveolar tissue damage in a variety of pulmonary pathologies including cystic fibrosis, asthma, transfusion-related acute lung injury and infections [8]. NETs may also provide the basis for biofilm formation, enabling the proliferation of resistant pneumococci, thereby contributing to pathologies such as otitis media (middle ear infection) [15], [16]. Consequently, the desire has been voiced to ameliorate the severity of these diverse conditions by pharmacologically modulating the NETotic process [3], [6], [8], [17], [18].

The signalling cascade triggering NETosis is known to involve several key steps, including the generation of reactive oxygen species (ROS) by NADPH oxidase, the translocation of the granular enzymes neutrophil elastase (NE) and myeloperoxidase (MPO) to the nucleus, where in concert with the citrullinating activity of peptidyl arginine deiminase type IV (PADI4) on histones, they promote chromatin decondensation [2], [19]–[21]. The upstream events appear to involve calcium flux, as NETs can be induced by calcium ionophores or by treatment with thapsigargin, which raises intracellular calcium stores by reducing calcium retention in the endoplasmic [7], [22], [23]. The action of calcium flux does not seem to be restricted to the generation of ROS, but also promotes histone citrullination by PADI4, a pivotal step in the NETotic process [22], [23]. The activation of protein kinase C (PKC) by phorbol ester (PMA) has also been shown to be important, and appears to depend on phosphorylation of p38 MAPK and ERK, by a pathway that may suppress apoptosis to permit NETosis [23]–[26].

An intriguing feature of these studies is that a considerable interplay appears to occur between the various signal-transducing elements during NETosis [23]. The extent of this interaction is largely determined by the initiating stimulus, in that the requirement for either NADPH oxidase or MPO activity may vary, depending on whether the initiating signal was provided by a physiological stimulus such as bacteria, or pharmacologically via PMA or ionomycin treatment [27]. In this regard, PMA was shown to suppress ionomycin induced histone citrullination [23].

As a physiological stimulus we used the inflammatory cytokine interleukin 8 (IL-8), which we have previously shown to play a potential role in aberrant NETosis associated with preeclampsia [4]. A number of other reports support the premise that IL-8 is able to activate neutrophils and induce NETosis [1], [4], [28], [29]. The action of IL-8 involves calcium flux via the mobilization of calcium from intracellular stores and influx of extracellular calcium, as well as PKC activation via G protein coupled receptors (GPCR) [18], [30], [31]. In our study, the action of IL-8 on these pathway components was compared to pharmacological stimuli mediated by PMA or ionomycin.

In our examination we specifically examined the roles of both the intracellular and extracellular calcium pools. Although these have been implicated in the efficient generation of ROS by NADPH oxidase [32], their contribution to NETosis has not been investigated in detail to date. This aspect was addressed by chelation of extracellular stores with EGTA (ethylene glycol tetraacetic acid), the chelation of intracellular stores with BAPTA-AM, or by inhibiting the mobilization of these with TMB-8 ([8-(N, N-diethylamino) octyl-3, 4, 5-trimethoxybenzoate]-hydrochloride) as described previously [30], [33], [34].

The clinical modulation of the calcium pathway in T-lymphocytes by calcineurin antagonizing molecules such as cyclosporine A (CsA) has powerful immune-suppressive properties [35]. This action, is however, not restricted to T cells, and a number of studies have indicated that the calcineurin pathway is functional in human neutrophils [36]–[38], and that its modulation by CsA affects a diverse array of neutrophil activities, including chemotaxis and phagocytosis [39]–[44].

To address the role of calcineurin in IL-8 induced NETosis, we investigated whether treatment with ascomycin and CsA influenced the NETotic process. As a control for the immunosuppressive activity of these drugs which target members of the immunophilin family, we used rapamycin, which targets the mTOR pathway [45].

The role of the G protein coupled receptor phospholipase C (GPCR-PLC) pathway was examined using pertussis toxin (PTX), an inhibitor of G protein coupled receptors, and U73122, an aminosteroid inhibitor of phospholipase C (PLC), as well as staurosporine (STS), an inhibitor of protein kinase C, using conditions similar to those described previously [46], [47].

## Materials and Methods

### Materials

Recombinant interleukin-8 (rIL-8), phorbol-12-myristate-13-acetate (PMA), dimethyl sulfoxide (DMSO), the extracellular calcium chelator EGTA (ethylene glycol tetraacetic acid), pharmacologic agents ascomycin and CsA to block calcineurin dependent signals, rapamycin, an inhibitor of mTOR, the GPCR inhibitor PTX, the PLC inhibitor U73122 [1-(6-(17β-3-methoxyestra-1,3,5(10)-trien-17-yl) amino)hexyl)-1H-pyrrole-2,5-dione], and the NADPH oxidase inhibitor DPI (diphenyl iodide) and 20% Dextran were purchased from Sigma (Sigma Chemicals, St. Louis, MO, USA). The intracellular calcium antagonist TMB-8 was purchased from Calbiochem (Nottingham, UK). All cell culture media and additives, Fluo-3 AM (fluo-3-acetoxymethyl ester), SYTOX Green and BAPTA-AM, an intracellular calcium chelator, were purchased from Invitrogen Life Technologies (Grand Island, NY, USA). Ficoll Paque plus was from Amersham (Amersham Biosciences, Uppsala, Sweden) and RBC lysis solution from Qiagen (Qiagen, USA).

### Isolation of neutrophils

Blood samples (20 ml each) for peripheral neutrophil isolation were obtained from healthy donors at the blood donation center of the Swiss Red Cross, University Hospital of Basel. Informed, written consent was obtained from all subjects in the study, which was approved by the Cantonal Ethical Review Boards of Aargau-Solothurn and Basel/Basel-Land, Switzerland. Neutrophils were isolated with previously established dextran-Ficoll methods [1], [4]. Neutrophil activation was examined by measuring CD11b up-regulation using flow cytometry to confirm that neutrophils were not activated during isolation (data not shown). In brief, mononuclear cells were depleted from whole blood by centrifugation over a Ficoll Paque plus gradient. The neutrophil-containing red blood cell (RBC) layer was first resuspended in HBSS (Hank's Balanced Salt Solution) medium, after which 20% Dextran was added to a 1% final concentration. The RBCs were then allowed to settle for 1 h at room temperature (RT), following which the neutrophil-containing upper layer was collected. The remaining RBCs were lysed for 5 min at RT using RBC lysis solution, and the neutrophil population (∼4–5×10^7^ cells) was obtained after washing twice with HBSS and resuspension in cell culture medium. The culture medium comprised of RPMI 1640 medium supplemented with 2% heat inactivated human serum, 2 mM L-glutamine, 100 U/ml penicillin 100 µg/ml and streptomycin. Trypan blue dye exclusion indicated that cell viability was 95–97% after the isolation. The purity of the isolated neutrophils was routinely >90%, as confirmed by flow cytometric analysis.

### Quantification of NETs release from activated neutrophils

NETs were quantified using fluorimetry as described previously [1], [4], [7], [28], [48]. Briefly, freshly isolated neutrophils (1×10^5^ cells) were cultured in 96-well microtitre plates and stimulated either with PMA at concentrations ranging between 10–50 nM, rIL-8 (25–100 ng/ml), or ionomycin (1–5 µM) for the indicated periods, as described previously [4], [7]. After the incubation, SYTOX Green, a non cell-permeant DNA binding dye, was added to the cells at 5 µM concentration to detect extracellular DNA. Unstimulated neutrophils were used as controls. The degree of the fluorescence of treated cells was obtained after subtracting the baseline fluorescence of unstimulated cells. The plates were read in a SpectraMAX Gemini fluorescence microplate reader (Molecular Devices, Sunnyvale, CA, USA) with a filter setting of 485 (excitation)/527 (emission) and the data were analysed using Soft Max Pro Software (Molecular Devices, Sunnyvale, CA, USA). The data were normalized as 100% NETs formation, either to the stimulus leading to the greatest degree of NETosis (e.g. PMA or Ionomycin), or for a given stimulus/pharmacological combination, in order to more readily assess the fold reduction (e.g. response to ascomycin, CsA, rapamycin in PMN stimulated with IL-8, PMA or ionomycin).

To ensure that cells were viable during the incubation period and that dead cells by apoptosis or necrosis did not contribute to an increase in fluorescence, cell viability was monitored using the WST-1 assay. This indicated that at least 90% of the cells were viable after 4 hours of treatment, which is considerably greater than the 1 hr timeframe most of our experiments were conducted in.

### Scanning electron and fluorescence microscopy of NETs

For scanning electron microscopy isolated neutrophils (1×10^5^ cells) were seeded on 12 mm 0.001% polylysine coated coverslips and incubated with the activators for 60 min and were fixed with 2.5% glutaraldehyde (Sigma Chemicals, St. Louis, MO, USA), post-fixed using repeated incubations with 1% osmium tetraoxide/1% tannic acid (Sigma Chemicals, St. Louis, MO, USA), and dehydrated with a graded ethanol series (30%, 50%, 70%, 100%). After dehydration and critical-point drying, the specimens were coated with 2 nm platinum and analysed with a Philips XL-30 ESEM scanning electron microscope at ZMB, Biozentrum, University of Basel [4], [7].

For fluorescence microscopy of NETs, neutrophils (5×10^5^ cells) were seeded in 24 well plates and were activated either with rIL-8 (100 ng/ml), PMA (50 nM), ionomycin (5 µM), if concentrations not indicated otherwise or left un-stimulated for 1 h. Then, 5 µM SYTOX Green dye was added to the culture and cells were incubated for 10 minutes. After this incubation, NETs were visualized using a Zeiss Axiovert fluorescence microscope and a Nikon Digital camera [4], [7].

### Immunohistochemical staining of NETs

5×10^4^ isolated PMNs seeded on poly-L-lysine-coated glass coverslips (BD Biosciences) in tissue-culture wells and allowed to settle prior to stimulation as described above. Coverslips were rinsed with ice-cold HBSS and the cells fixed with 4% paraformaldehyde and blocked overnight (HBSS with 10% goat serum, 1% BSA, 0.1% Tween20, and 2 mM EDTA) at 4°C. NETs were detected with rabbit anti-MPO (Dako) and rabbit anti-citrullinated histone H3 (citH3, Abcam). Secondary antibodies were goat anti-rabbit IgG AF555 and goat anti-rabbit IgG AF488 (Invitrogen). DNA was stained with 4',6-diamidino-2-phenylindole (DAPI, Sigma) and NETs were visualized using a Zeiss Axioplan 2 Imaging fluorescence microscope in conjunction with a Zeiss AxioCam MRm monochromatic CCD camera and analyzed with Axiovision 4.8.2 software (Carl Zeiss).

### Measurement of intracellular calcium

Fluo-3 AM was used as the fluorescent, Ca^2+^-sensitive indicator for these experiments [49]. 1×10^7^ neutrophils were loaded with 5 µM Fluo-3 AM in RPMI medium for 45 min at 37°C. The cells were then washed with PBS twice to remove extra Fluo-3 AM. An aliquot of Fluo-3 AM-loaded neutrophils was resuspended in RPMI medium at the concentration of 1×10^6^ cells/ml. The cells were then transferred to a 6 well plate maintained at 37°C, and the fluorescence emission was recorded using a spectrofluorimeter (Molecular devices, Sunnyvale CA, USA). Excitation and emission fluorescence were at 506 and 526 nm, respectively. Cellular auto-fluorescence accounted for nearly 10% of the total signal.

For the calibration procedure, maximum fluorescence was determined by the disruption of the cells with 1% Triton x-100 and the addition of 10 mM EGTA. The calcium concentration was calculated as described previously, using a dissociation constant for Ca^2+^-bound Fluo-3 of 400 nmol/l [49]. For this experiment, neutrophils were activated with either 50 nM PMA or 100 ng/ml IL-8 and release of intracellular Ca^2+^ was monitored for up to 5 minutes. 1 µM ionomycin was used as a positive control for measuring release of intracellular calcium.

### Examination of intracellular calcium flux in activated neutrophils using fluorescence microscopy

Detection of calcium flux by fluorescent microscopy was carried out as described previously [30]. In brief, neutrophils were isolated from 20 ml blood. Cells were resuspended at 1×10^7^ cells/ml concentration in the HBSS medium and were loaded with 5 µM Fluo-3 AM at 37°C for 30 min. Cells were washed twice with HBSS at 800 g for 10 min each and resuspended in RPMI (complete medium) at 1×10^6^ cells/ml concentration. 500 µl of the cell suspension (5×10^5^ cells) was plated on polylysine coated slides and the cells were allowed to adhere for 10 min. Selected activators were added at required concentrations and cells were incubated at 37°C for 30 min in a humidified chamber. After the incubation samples were fixed for 60 minutes with 4% PFA and washed twice with PBS for 5 minutes each time. Before DAPI staining cells were treated with RNAse (1 mg/ml in PBS) for 20 minutes in the dark. Slides were washed twice in PBS for 5 minutes each, air dried and mounted with fluorophore mounting medium with DAPI. Cells were analyzed using a Zeiss Axioplan 2 imaging fluorescent microscope.

### Influence of calcium sequestration on NETosis

To assess the role of intra- and extracellular calcium pools on NET formation, 1×10^5^ cells were cultured in 96 well plates in RPMI 1640 complete medium supplemented with 2% heat inactivated human serum, 2 mM L-glutamine, 100 U/ml penicillin, and 100 µg/ml streptomycin. The intracellular calcium antagonist TMB-8 (20 µM) and extracellular calcium chelator EGTA (10 mM) were used to sequester calcium or prevent calcium release. Cells were pre-incubated with TMB-8 for 15 minutes and washed prior to further treatment. NET formation was monitored in normal calcium rich media or that containing EGTA, TMB-8 or both using fluorimetry as described above. In addition, intracellular calcium was chelated using cells pre-incubated for 15 minutes with BAPT-AM, and subsequently washed prior to further stimulation with IL-8, PMA or ionomycine. This was done to prevent any chelation of extracellular calcium by this agent. The conditions chosen were analogous to those reported previously [30].

### Analysis of NETs formation after treatment with pharmacological agents

To assess the influence of pharmacological agents on NETosis, with specific regard to NETs induction by IL-8 generation, drugs were always added 15 minutes prior to the activation of cells, after which the cells were washed, and incubated for the indicated time and doses. NET formation was assessed using fluorescent microscopy and quantification was carried out using spectro-fluorimetery after the addition of SYTOX Green dye (as described above). The viability of the cells during all pharmacological studies was greater than 90% using the trypan blue dye exclusion test, or when monitored with the WST-1 viability assay. For these diverse experiments conditions were chosen similar to those reported previously [30], [40], [41], [46], [47].

### Chemiluminescence assay for NADPH oxidase activity

Measurement of NADPH oxidase mediated ROS production was performed in HBSS media containing 2 mM HEPES, 2% heat inactivated human serum and 10 µM luminol (5-amino-2,3-dihydro 1,4-phthalazinedione). Luminol is a membrane-permeable molecule that reacts within secreted reactive oxygen species (ROS) as well as those produced in an intracellular compartment (granules or phagosomes) [31]. Briefly, 5×10^5^ cells/well were incubated in HBSS medium. The baseline was measured in a thermostatically controlled Berthold LB96V luminometer (Berthold Technologies, Bad Wildbad, Germany) set at 37°C. The respiratory burst was then stimulated with 50 nM PMA, 100 ng/ml IL-8 or 5 µM ionomycin with or without DPI or EGTA or EGTA+TMB-8. The response was recorded over a period of 15 minutes. The luminescence was measured as relative light unit seconds (RLU.sec).

### Monitoring of cell viability by WST-1 assay

Viability of the neutrophils following pharmacological treatment was assessed using the commercial WST-1 (Roche diagnostics, Switzerland) assay as per the manufacturer's instructions. Briefly, 1×10^5^ purified neutrophils were seeded in 96 well plates in 100 µl complete RPMI medium. Cells were pre-treated with pharmacological antagonists (e.g. ascomycin, cyclosporine A) for 15 minutes, following which activators such as PMA, IL-8 or ionomycin were added to the cell culture. Immediately after the addition of the activators 10 µl of the WST-1 solution was added to each well to check viability of the cells by assessing for the presence of metabolically active cells. Plates were incubated at 37°C in a tissue culture incubator for up to 4 hrs. Cell viability was measured at the indicated time points in a microplate reader (absorbance 450 nm) (Molecular Devices, Sunnyvale, CA, USA) and the data was analysed using Soft Max Pro Software (Molecular Devices, Sunnyvale, CA, USA). Absorbance obtained from the freshly isolated untreated cells was considered as 100% viable cells.

### Statistical analysis

Data were analysed using GraphPad Prism 6 for Mac OSX. The unpaired t-test was used to calculate two-tailed P value to estimate statistical significance of differences between two groups. One- or two-way ANOVA with Bonferroni correction were applied to assess differences between multiple groups. Statistically significant P values were indicated in the figures as follows: ****, P<0.0001, ***, P<0.001, **, P<0.01 and *, P<0.05. Intra and inter group comparisons are indicated by bar ends.

## Results

### Role of intracellular and extracellular calcium flux in NETosis

**Figure 1:**
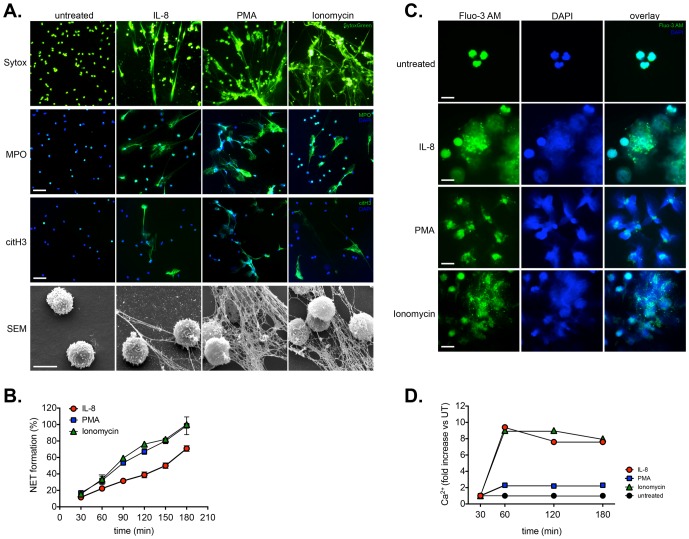
Detection of IL-8 induced NETosis and calcium mobilization. **A. Upper panel**: Detection of NETs induced by IL-8, PMA or ionomycin by fluorescent microscopy using SYTOX Green. Magnification: 20x. **Middle panel**: Detection of NETs induced by IL-8, PMA or ionomycin by fluorescent immunohistochemistry for the presence of MPO (myeloperoxidase) or citrullinated histone H3 (citH3) on NETs structures. Magnification: 20x; scale bar: 50 nm **Lower panel**: Detection of NETs induced by IL-8, PMA or ionomycin scanning electron microscopy (SEM). NETs were induced by treatment with rIL-8 (100 ng/ml), PMA (50 nM) or ionomycin (5 µM). Scale bar: 10 µm. **B. Quantification of NETs release from activated neutrophils by fluorimetry.** NETs were induced by treatment with rIL-8 (100 ng/ml), PMA (50 nM) or ionomycin (5 µM) and monitored over a period of 180 minutes. NET formation (100%) is normalized to that mediated by ionomycin (5 µM) or PMA (50 nM) treatment after 180 minutes, indicating a reduced NETotic response triggered by IL-8 (ng/ml). **C. Examination of intracellular calcium release in activated neutrophils by fluorescence microscopy.** Detection of calcium mobilization by fluorescent microscopy using Fluo-3 AM loaded neutrophils. Mobilization of intracellular calcium was noted in cells treated with ionomycin or IL-8, while only minimal flux was detected in PMA treated cells. Magnification: 40x; scale bar: 20 nm. **D. Quantitation of intracellular calcium levels.** Fluorimetric quantitation of intracellular calcium flux using Fluo-3 AM loaded neutrophils. Both IL-8 and ionomycin treatment lead to substantial increases in free intracellular calcium, unlike PMA, where only a minimal increase was noted.

We, and others, have previously observed that treatment with IL-8 induced NETs generation in isolated PMN [1], [4], [7], [28] (refer to Figure 1A). Since IL-8 leads to calcium mobilization in neutrophils, we were intrigued by recent reports indicating that that solitary treatment with calcium ionophores such as ionomycin can trigger NETosis [22], [27], especially as it had not been determined what the relative contribution by either external or intracellular calcium pools was.

To investigate this aspect we examined NETosis induced by IL-8, the phorbol ester PMA and ionomycin; the presence of NETs being detected by fluorescent microscopy for SYTOX Green staining, as well as scanning electron microscopy (SEM) (refer to Figure 1A and supplementary video S1 and S2). In addition, we performed fluorescent immunohistochemistry for NETs associated granular proteins (myeloperoxidase, MPO), or post-translational histone modifications (citrullinated histone H3, citH3), shown to play a role in the NETotic process (Figure 1A).

This facet is based on the finding that NETosis relies on the action of peptidyl arginine-deiminase 4 (PADI4) in the nucleus, where it citrullinates histones, thereby promoting chromatin decondensation [20], [22], [50]. In combination with the action of neutrophil elastase (NE) and myeloperoxidase (MPO), these steps facilitate the extrusion of nuclear DNA into the extracellular environment [19], [51]. The occurrence of such an event can be shown by presence of citrullinated histones in association with either MPO or NE on the external NETs structures [13], [22], [52].

Our data, therefore, confirm the occurrence of NETs structures by this pathway following IL-8 treatment, as fluorescent immunohistochemistry indicates that these NETs contain MPO as well as citrullinated histones (CitH3), in association with DNA (DAPI) (Figure 1A).

These data also indicated that IL-8 induced NETosis, but that it was not as pronounced as that exerted by high doses of the pharmacological agents PMA and ionomycin, as is also evident in the supplementary time-lapse microscopy videos (supplementary video S1 and S2).

Since the quantification of NETosis using a variety of stimuli, inhibitors or kinetic conditions is time consuming, labour intensive and difficult to assess quantitatively using microscopy-based approaches [53], we elected to use a fluorescent approach in which extruded NETs were quantified by their binding to SYTOX Green, which we and other have used on previous occasions for this purpose [1], [4], [7], [28], [48], [54]. By these means we were able to follow the kinetics of NETosis triggered by IL-8, PMN and ionomycin (refer to Figure 1B). Once again, NETosis induced by IL-8 was determined to be slower and less pronounced that that induced pharmacologically by PMA or ionomycin.

To ensure that our chosen stimulus, IL-8, did indeed lead to calcium mobilization, we assessed the release of intracellular calcium using Flou-3 AM loaded neutrophils by fluorescent microscopy (refer to Figure 1C). This analysis indicated that release of intracellular calcium was readily evident following IL-8 and ionomycin treatment, whilst it was minimal following PMA treatment (refer to Figure 1C).

A quantitative fluorimetric analysis indicated that IL-8 and ionomycin treatment lead to a 7–8 fold increase in intracellular calcium levels, while PMA treatment only triggered a minimal increase in calcium levels (Figure 1D), thereby corroborating the fluorescent microscopy observations.

To evaluate dependency of NETs formation on either the mobilization of extra- or intracellular calcium pools, we chelated extracellular calcium with EGTA, antagonized intracellular mobilization with TMB-8, or combinations of both and monitored NETs generation in response to IL-8 and PMA.

**Figure 2:**
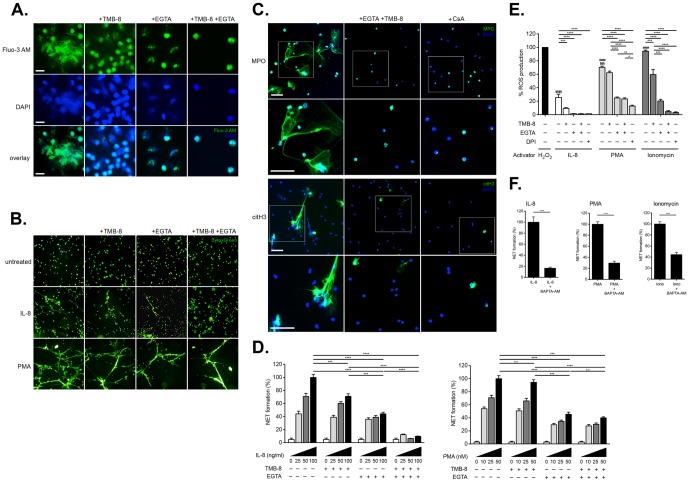
Role of intra- and extracellular calcium pools in the induction of NETosis by IL-8. A. Examination of intracellular calcium mobilization by fluorescence microscopy in IL-8 stimulated neutrophils treated with TMB-8 and EGTA. Calcium mobilization was triggered by treatment with IL-8. Intracellular calcium utilization was antagonized by TMB-8, while extracellular calcium was chelated with EGTA. Mobilization of intracellular calcium in cells treated with IL-8 was greatly reduced by chelation of external calcium by EGTA or binding of internal stores by TMB-8, and largely abolished by both agents in combination. Magnification: 40x; scale bar: 20 nm. **B. Detection of NETosis by fluorescent microscopy using SYTOX Green under calcium sequestering conditions.** NETosis induced by IL-8 is greatly reduced by EGTA, to a lesser extent by TMB-8, and completely abolished by the action of both agents. NETosis triggered by PMA was largely unaffected by the action of TMB-8, being more sensitive to the chelation of extracellular calcium by EGTA. Magnification: 10x. NETs generation was normalized as 100% for 10 ng/ml IL-8 or 50 nM PMA treatment. **C. Detection of NETs by fluorescent immunohistochemistry following calcium sequestration or treatment with cyclosporine A.** The combined action of TMB-8 and EGTA effectively blocked IL-8 induced NETs as detected by the presence of citH3 or MPO. In a similar manner, treatment with the calcineurin antagonist CSA, greatly diminishes IL-8 induced NETs generation. Magnification: 20x; scale bar: 50 nm. Inserts indicate higher magnification view in panel immediately below. **D. Quantitative fluorimetric detection of IL-8 or PMA induced NETosis following calcium sequestration.** Upper panel illustrates NETosis induced by IL-8, while lower panel illustrates NETosis induced by PMA. For this analysis, neutrophils (1×10^5^/well) were plated in 96-well plates in the appropriately modified medium and activated with increasing doses IL-8 (0–100 ng/ml). Following 1 hr culture, NETs were quantified fluorimetrically and the data normalized as described in Materials and Methods. An analysis from 4 different experiments is shown, and the data are presented as mean ± SEM. Two-way ANOVA with Bonferroni correction were applied to assess differences between multiple groups. Statistically significant P values are indicated in the graphs as follows: ****, P<0.0001 and ***, P<0.001. **E. Influence of calcium sequestration on ROS production.** ROS was measure via Luminol detection after induction with rIL-8 (100 ng/ml) or PMA (50 nM) or ionomycin (5 µM). H_2_O_2_ (50 µM) served as a relative control and considered 100%. DPI (25 µM), a specific inhibitor of NADPH mediated ROS generation, was used to block ROS production and was used as negative control. Extracellular calcium was chelated with EGTA and release of intracellular calcium was antagonized with TMB-8. An analysis from 6 different experiments is shown, and the data presented as mean ± SD (standard deviation). Two-way ANOVA with Bonferroni correction were applied to assess differences between multiple groups. Statistically significant P values are indicated in the graphs as follows: ****, P<0.0001; ***, P<0.001; **, P<0.01 and *, P<0.05. **F: Chelation of intracellular calcium reduces NETosis.** Neutrophils (1×105/well) were plated in the 96-well plates in triplicates. Cells were pre-treated with 10 µM BAPTA-AM, a cell permeable calcium chelator, for 15 minutes, washed extensively and then activated with either 100 ng/ml IL-8, or with 50 nM PMA, or with 5 µM ionomycin. NETs were quantified fluorimetrically following 1 hour culture as described. An analysis from 8 different experiments is shown, and the data are presented as mean ± SD (standard deviation). Unpaired t-test was used to calculate two-tailed P value to estimate statistical significance of differences between two groups. Statistically significant P values are indicated in the graphs as follows: Statistically significant P values are indicated in the graphs as follows: ****, P<0.0001. The data were normalized as 100% NET formation as induced by treatment with rIL-8 (100 ng/ml), PMA (50 nM) or ionomycin (5 µM) in the absence of any inhibitors.

Fluorescent microscopy of Flou-3 AM loaded neutrophils indicated that TMB-8 or EGTA greatly reduced calcium mobilization by IL-8, and that it was completely abolished by the use of both reagents in combination (Figure 2A).

An analysis of NETs generation detected by SYTOX Green staining and fluorescent microscopy, indicated that that TMB-8 reduced IL-8 induced NETosis, but not to the same extent as the effect mediated by EGTA or both agents in combination (Figure 2B). The induction of NETosis by PMA, on the other hand, was not greatly influenced by TMB-8, and while it was reduced by the action of EGTA, it was not completely abrogated by both agents combined (Figure 2B).

To further confirm the influence of calcium chelation on IL-8 induced NETosis, we examined for the presence of NETs as detected by fluorescent immunohistochemistry for MPO and citrullinated histone H3 (Figure 2C). This experiment indicated that the combined action of TMB-8 and EGTA greatly diminished IL-8 induced NETosis, as is evident by the absence of appropriately immunohistochemically stained NETs structures (Figure 2C).

When this aspect was monitored by fluorimetric analysis it was observed that in normal calcium rich medium there was a dose dependent increase in NETs upon stimulation with IL-8 (Figure 2D, left panel). However, when intracellular calcium release was blocked with TMB-8 or extracellular calcium was chelated with EGTA, a significant reduction in NETs formation was observed (Figure 2D, left panel). When flux from both intracellular and extracellular calcium pools was restricted by the combined use of EGTA and TMB-8, NET formation triggered by IL-8 was almost completely inhibited (Figure 2D, left panel). These data therefore parallel the observations made using fluorescent microscopy (Figure 2B, 2C).

On the other hand, as was evident from the fluorescent microscopic analysis (Figure 2B), the quantitative fluorimetric monitoring of NETs induction by PMA under similar culture conditions indicated that this was only sensitive to chelation of extracellular calcium using EGTA (Figure 2D, right panel).

These two independent data sets therefore suggest that IL-8 mediated NETs formation requires calcium fluxes from both intracellular and extracellular pools, while that primarily extracellular calcium is required for efficient PMA mediated NET generation.

To examine by which means this calcium sequestration inhibited IL-8 induced NETosis, we examined ROS production by NAPDH oxidase using a Luminol chemiluminescent assay [31]. This analysis indicated that the action of TMB-8 reduced IL-8 mediated ROS production, that this was significantly reduced by the action of EGTA and almost completely abolished by the combined action of TMB-8 and EGTA (refer to Figure 2E). As expected ROS production was completely prohibited by DPI, a NADPH oxidase antagonist (Figure 2E). These data also indicated that PMA induced ROS produced was largely unaffected by the action of TMB-8, and was reduced by approximately 60% by the action of EGTA (Figure 2E). This latter level was, however, still almost as high as that induced by IL-8 in the absence of any calcium chelating agents (Figure 2E). This data also confirmed that treatment with ionomycin induced ROS production by NADPH oxidase, and that this diminished by the combined action of TMB-8 and EGTA and abolished by DPI treatment (Figure 2E).

### Intracellular chelation of calcium reduced NETs formation

Our data, hence, suggest that NET generation induced by IL-8 involves both intra- and extracellular calcium pools, while PMA-mediated NET induction involved influx of extracellular calcium.

To further corroborate the involvement of calcium in IL-8 induced NETosis, we performed an additional analysis using BAPTA-AM, a chelator of intracellular calcium. By this means signals mediated either by the trans-membrane passage of extracellular calcium or the release from intracellular stores, would be blocked. In this experiment, the cells were pre-incubated with BAPTA-AM for 15 minutes and extensively washed prior to treatment with IL-8 or PMA. In this manner any chelation of extracellular calcium by BAPTA-AM was minimized.

The results of this experiment confirmed that chelation of intracellular calcium with BAPTA-AM leads to a significant reduction in IL-8 mediated NET generation, and to a lower extent in cells treated with PMA or ionomycin (Figure 2F).

### Treatment with CsA or ascomycin diminishes NETs formation

Pharmacological inactivation of calcineurin, a calcium-dependent serine/threonine protein phosphatase, is an important immunosuppressive mechanism [35]. In this context active calcineurin has previously been detected in human neutrophils [36], [39], [55], as well as other members of the signalling cascade such as NFAT2 (nuclear factor of activated T cells 2) [37]. In addition a number of previous studies have indicated calcineurin inhibition significantly influenced neutrophil activity, inhibiting chemokinesis, adhesion and motility on vitronectin, response to angiotensin II, as well as a reduction in phagocytic activity [38]–[41], [43], [44].

These diverse reports suggest that the calcineurin pathway is functional in human neutrophils and that its activity can be modulated by antagonists such as CsA.

For this reason we investigated whether inhibition of calcineurin modulates NETosis. Evidence of such an interaction was obtained from a pilot experiment, in which we examined the effect of cyclosporine A (CsA) on IL-8 induced NETosis, using fluorescent immunohistochemistry for citrullinated histone H3 (citH3), MPO and DNA (DAPI) (Figure 2C). This experiment indicated that CSA treatment reduced the extent of IL-8 induced NETosis (refer to Figure 2C).

As these data suggest that CsA antagonizes the action of IL-8 in triggering NETosis, we explored this facet in greater detail, for which purpose we used two other well characterized immunosuppressant drugs, namely ascomycin (an analogue of FK506/tacrolimus) and rapamycin.

CsA and ascomycin inactivate calcineurin phosphatase activity by engaging with the immunophilins cyclophilin A and macrophilin-12, respectively [35], while rapamycin targets mTOR rather than calcineurin [45]. To explore these in detail we used a fluorimetric detection of NETs generation [1], [4], [7], [28], [48], [54].

**Figure 3:**
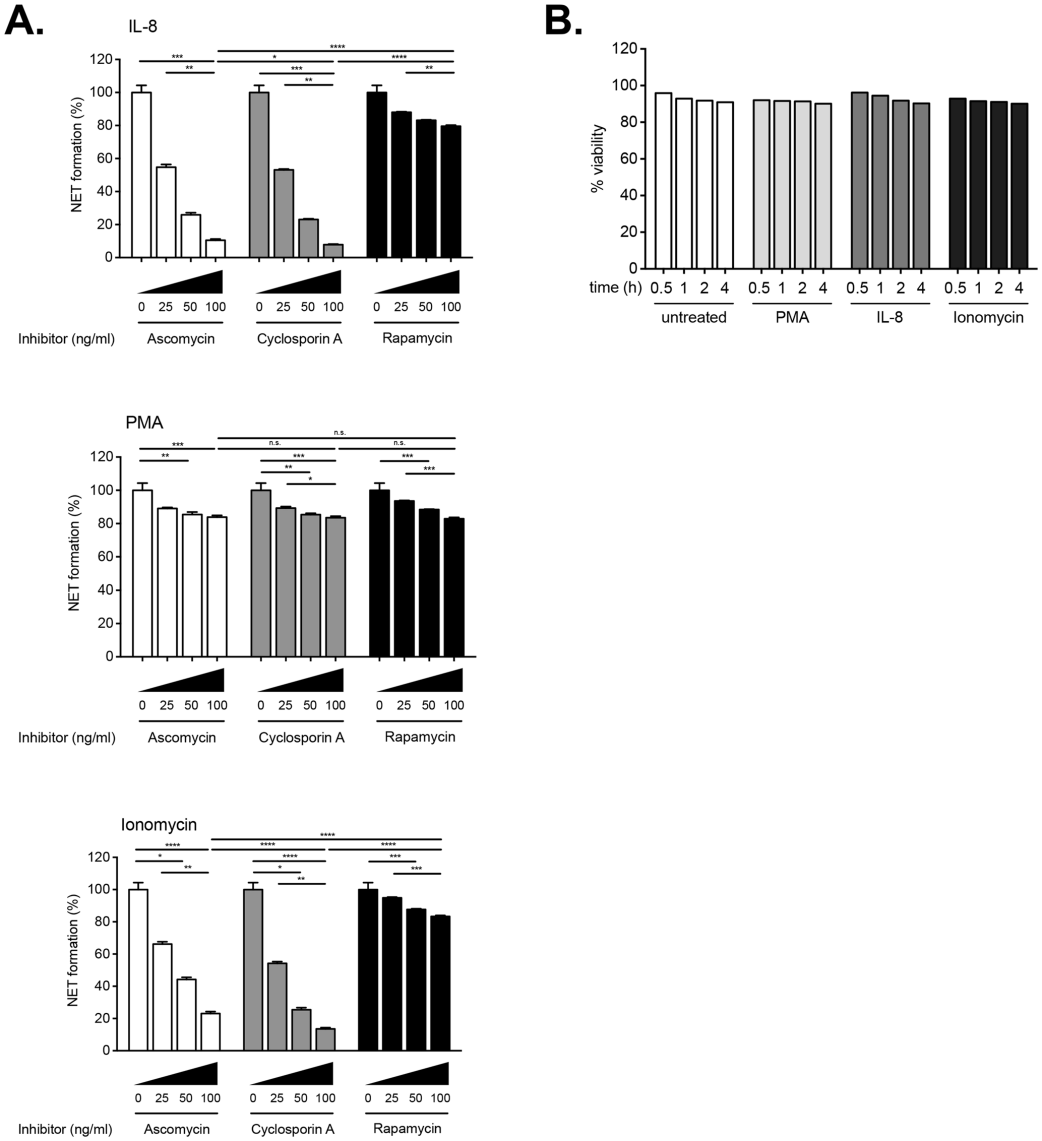
Inhibition of IL-8 induced NETosis by ascomycin and cyclosporine A. A. Quantitative fluorimetric detection of NETosis modulated by ascomycin, cyclosporine A or rapamycin. 1×10^5^ cells/well were plated in triplicates in 96-well plates. Cells were pre-incubated with the indicated increasing doses (0–200 ng/ml) of calcineurin antagonists for 15 min at 37°C prior to the addition of 100 ng/ml IL-8 (upper panel), or 50 nM PMA (middle panel), or 5 µM ionomycin (lower panel). NETs were quantified fluorimetrically after 1 hour culture using SYTOX Green dye. An analysis from 8 different experiments is shown, and the data presented as mean ± SD (standard deviation). NET formation was normalized to 100% as induced by treatment with rIL-8 (100 ng/ml), PMA (50 nM) or ionomycin (5 µM) in the absence of any inhibitors. Two-way ANOVA with Bonferroni correction were applied to assess differences between multiple groups. Statistically significant P values are indicated in the graphs as follows Statistically significant P values are indicated in the graphs as follows: ****, P<0.0001 and *, P<0.05. **B. Monitoring of cell viability by WST-1 assay.** In this representative experiment cells (1×10^5^ cells/well) were pre-treated with Ascomycin for 15 minutes, following which they were either left without further stimulus, or stimulated with 100 ng/ml IL-8, 50 nM PMA or 5 µM ionomycin. Following addition of the WST-1 reagent, cell viability was monitored over a period of 4 hours according to the manufacturer's instructions. This assay indicates that the vast majority of cells were metabolically active over the entire culture period. No significant changes were noticed between any of the groups.

These studies indicated that pre-incubation of neutrophils for 15 min with increasing doses of ascomycin or CsA greatly reduced NETs generation by IL-8 (Figure 3A, upper panel). The action of the two calcineurin antagonists similarly reduced NETosis induced by ionomycin (Figure 3A, middle panel), while in contrast, these drugs did not demonstrate any noticeable effect on PMA-mediated NETs generation (Figure 3A, lower panel).

Rapamycin did not reduce NETosis to the same extent as either CsA or ascomycin under of the conditions examined (Figure 3A, all panels). This result implies that that mTOR was not extensively involved in the signal transducing pathways triggered by either of the stimuli used.

Under the experimental conditions used, none of these immunosuppressant drugs was found to induce apoptosis in neutrophils as assessed by the trypan blue dye exclusion test, or when viability was monitored using the WST-1 assay. An example using ascomycin is illustrated in Figure 3B. Our data, therefore, suggest that the calcium signalling event triggered by IL-8 is transmitted via calcineurin for efficient NET induction.

### Involvement of the GPCR-PLC pathway

Since the action of IL-8 invokes the GPCR-PLC pathway [30], which modulates intracellular calcium concentrations via the action of IP_3_ (inositol 1,4,5-trisphosphate), we examined the action of PTX and U73122. PTX inhibits GPCR signalling, by catalysing ADP ribosylation of G-protein α subunits of the G*i* family, thereby preventing interaction with the G-protein coupled IL-8 receptor [30], [56]. Although PTX has previously been shown to influence neutrophil chemotaxis [46], it has recently been shown that *Bordetella pertussis* promotes secondary infections by suppressing the innate immune response [57], [58]. Hence, it is of considerable interest to examine whether PTX influences the NETotic process.

**Figure 4:**
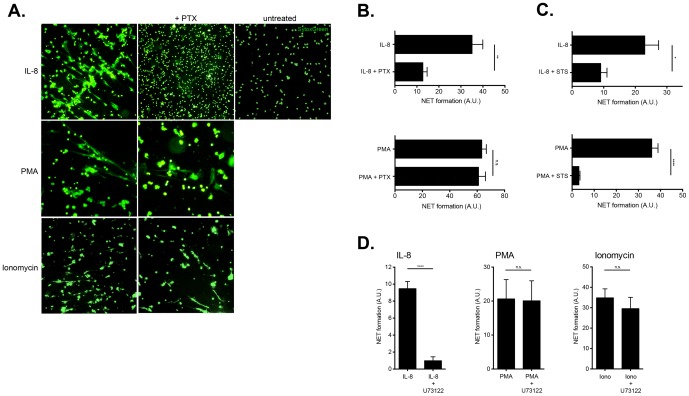
Involvement of the GPCR-PLC pathway in IL-8 induced NETosis. A. Treatment with pertussis toxin inhibits IL-8 induced NETosis. Isolated neutrophils were pretreated with pertussis toxin (PTX) (100 ng/ml) for 15 minutes and then cultured with activators, rIL-8 (100 ng/ml), PMA (50 nM), ionomycin (5 µM) for 1 hour. NETosis was detected by fluorescent microscopy for SYTOX Green stained extracellular DNA structures. Magnification: 10x. **B. Fluorimetric detection of NETosis reduction by pertussis toxin treatment in IL-8 stimulated neutrophils.** This figure illustrates a quantitative reduction in IL-8 induced NETosis by treatment with PTX (15 minute pre-treatment) (upper panel), while that triggered by PMA (lower panel), was largely unaffected. An analysis from 6 different experiments is shown, and the data presented as mean ± SD (standard deviation). NET formation was normalized to 100% as induced by treatment with rIL-8 (100 ng/ml) or PMA (50 nM) in the absence of any inhibitors. Unpaired t-test was used to calculate two-tailed P value to estimate statistical significance of differences between two groups. Statistically significant P values are indicated in the graphs as follows: **, P<0.01. **C. Influence of staurosporine treatment on IL-8 induced NETosis.** This figure illustrates a quantitative reduction in IL-8 induced NETosis by treatment with the protein kinase C inhibitor, staurosporine (STS) (15 minute pre-treatment) (upper panel), and almost complete abolition of that triggered by PMA. An analysis from 6 different experiments is shown, and the data presented as mean ± SD (standard deviation). NET formation was normalized to 100% as induced by treatment with rIL-8 (100 ng/ml) or PMA (50 nM) in the absence of any inhibitors. Unpaired t test was used to calculate two-tailed P value to estimate statistical significance of differences between two groups. Statistically significant P values are indicated in the graphs as follows: **, P<0.01. **D. Inhibition of Phospholipase C reduced IL-8 induced NETosis.** Following pre-treatment with U73122 for 15 minutes, an inhibitor of phospholipase C, neutrophils were cultured for 1 hour in the presence of 100 ng/ml IL-8, or 50 nM PMA, or 5 µM ionomycin. These data indicate that only IL-8 induced NETosis was significantly affected. NETs were quantified fluorimetrically after 1 hr culture using SYTOX Green dye and the data normalized as described. An analysis from 4 different experiments is shown, and the data presented as mean ± SD (standard deviation). Unpaired t-test was used to calculate two-tailed P value to estimate statistical significance of differences between two groups. Statistically significant P values are indicated in the graphs as follows: Statistically significant P values are indicated in the graphs as follows: ****, P<0.0001.

Our data using fluorescent microscopy for SYTOX Green stained extracellular NETs structures, indicate that PTX markedly reduces IL-8 induced NETs, while NETosis triggered by PMA or ionomycin is unaffected (Figure 4A).

To verify and quantify this reduction in NETosis, we used quantitative fluorimetry, which confirmed that IL-8 induced NETosis was significantly reduced by PTX treatment (Figure 4B, upper panel), while that triggered by PMA was largely not affected at all (Figure 4B, lower panel). These data therefore indicate the reliance of IL-8 receptor on functional G-protein interaction, while PMA and ionomycin bypass this step.

To confirm that PKC was involved in the signal transduction machinery employed by IL-8, we pre-treated isolated neutrophils with staurosporine (STS), a PKC inhibitor, whose action has previously been explored in human neutrophils [47]. These experiments indicated that staurosporine significantly reduced NETosis induced by IL-8, while it almost completely abolished that triggered by PMA (Figure 4C).

We also examined a further step in this signalling cascade, namely PLC, which can be inhibited by the action of U73122 [18], [30], [47]. Akin to what we observed for PTX, we determined that inhibition of PLC by U73122 reduced IL-8 induced NETosis, but not that mediated by PMA or ionomycin (Figure 4D).

These data, therefore, underline the importance of calcium mobilization for physiologically induced NETosis triggered by IL-8 and GPCR-PLC signalling.

**Figure 5:**
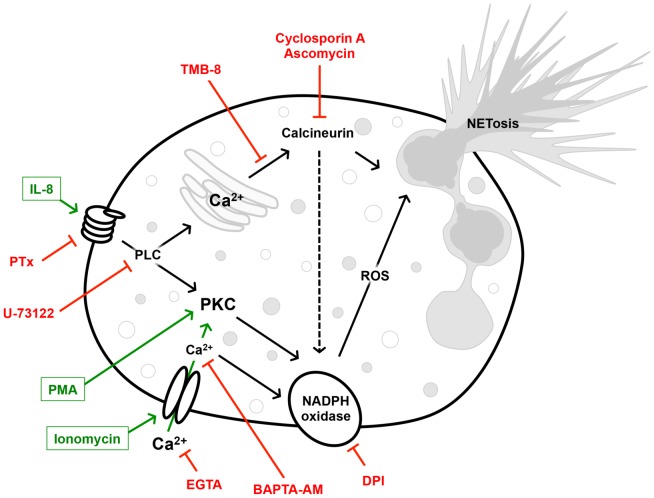
Schematic representation of the calcium-dependent signal transduction pathway triggered by IL-8. The action of calcineurin is antagonized by Cyclosporine A and Ascomycin, that of the G protein coupled IL-8 receptor by PTX and that of PLC by U73122. External calcium is chelated by the action of EGTA, while intracellular calcium is sequestered by the cell permeable chelator BAPTA-AM. The release of intracellular calcium from the endoplasmic reticulum is antagonized by TMB-8. DPI inhibits the action of the NADPH oxidase required for ROS production, a key step in NETosis.

A graphical summary of the potential signal-transducing cascade is provided in Figure 5.

## Discussion

NETosis has been shown to be dependent on the generation of ROS by activation of the NAPDH oxidase and histone citrullination by PADI4 activity, both of which are calcium dependent events [2], [20]–[22]. Since a number of recent studies have indicated that aberrant NETosis may play a role in human pathologies ranging from preeclampsia, systemic lupus erythromatosus, rheumatoid arthritis to coagulopathies or infectious conditions such as otitis media, the need for pharmacologic therapeutic intervention has been voiced [3], [6], [8].

As we had previously observed that placentally-derived IL-8 played a potential role in the triggering of aberrant NETosis in preeclampsia [4], we have now examined the role of calcium signalling in this process, and whether this pathway was subject to modulation by pharmacologic agents.

IL-8 transduces its signal in neutrophils by binding to the CXCR1, and, to a lesser extent to CXCR2, members of the G protein coupled family of serpentine (7 trans-membrane) receptors [18], [30], [31]. Both receptors are constitutively expressed on neutrophils.

Downstream calcium signalling induced by IL-8 in neutrophils occurs in two phases: a rapid initial release from intracellular stores, mainly from the endoplasmic reticulum via the activation of phospholipase Cβ and the subsequent action of IP_3_, followed by a prolonged influx mediated by the activation of Ca^2+^ release-activated Ca^2+^ (CRAC) channels [18], [30], [31].

Previous studies have indicated that the activation of neutrophils by IL-8 was sensitive to calcium chelation by EGTA, while TMB-8 was shown to limit release of calcium from intra-cellular stores [30], [33], [34].

By using analogous conditions, our data using EGTA and TMB-8, both of which lead to reductions in the number of NETs generated, especially when used in combination, indicate that both of these calcium sources are required for efficient NETosis. The importance of calcium signalling was additionally supported by the data obtained with BAPTA-AM, an intracellular chelator, whose action greatly diminished IL-8 induced NETosis.

It is well established that the classical G protein coupled receptor phospholipase C signalling pathway triggered by IL-8 is abrogated by PTX [29]. In our experiments we observed that the action of PTX greatly reduced NETosis, thereby confirming that PTX sensitive G protein subunits play a role in transducing the activating signal to phospholipase C in this signalling cascade (refer to Figure 5). As NETosis was not completely abolished by PTX treatment, it would appear that part of the signal to phospholipase Cβ is transmitted by G protein subunits that are not sensitive to pertussis toxin action.

An important facet that we explored in our study, with regard to calcium signalling, is whether NET formation could be modulated by pharmacologic agents, specifically those antagonizing the action of calcineurin, such as CsA or ascomycin [35]. This investigation was prompted by a number of reports indicating that human neutrophils possess a functional calcineurin system [36], [39], [55], including nuclear signalling components such as NFAT2 [37], and that its pharmacological modulation influences neutrophil behaviour [38]–[41], [43], [44].

By using analogous conditions to these reports, we observed that treatment with either CsA or ascomycin significantly reduced NETosis in a dose dependent manner, while it was largely unaffected by treatment with rapamycin, which utilizes the mTOR pathway.

These data, therefore, provide additional evidence of the role of calcium signalling in NETosis triggered by IL-8. In addition, since both CsA and ascomycin reduced ionomycin induced NETosis, this illustrates that calcineurin is downstream of the event mediating calcium liberation.

Such a premise is supported by previous studies which indicated that treatment of neutrophils with CsA decreased ROS production, but did not effect calcium liberation [41], [42], [59], thereby demonstrating CsA acts downstream of the receptor mediated event triggering calcium flux.

Of interest is that the interaction between calcium and calcineurin does not appear to be crucial for PMA induced NETosis, as this was not affected by CsA nor ascomycin treatment.

On the other hand, rapamycin, did not hinder NETosis, whether induced by IL-8, PMA or ionomycin, which excludes an involvement of the mTOR pathway in the signalling pathways activated by these agonists [45]. This result is intriguing since recent reports have indicated that rapamycin may influence NETosis in mTOR -dependent pathways involving autophagy or HIF-1α (hypoxia inducible factor 1α) [60], [61]. In addition, since PADI4 may influence mTOR expression, it is conceivable that rapamycin modulates the NETotic response under other conditions [62]. An overview of the potential signal transducing mechanism is provided in Figure 5.

The inhibitory action of the immunosuppressive agents CsA and ascomycin on NETosis may have clinical implications in offering a therapeutic target in conditions in which overt or aberrant NETosis contributes to the underlying pathology. In this context it is worth noting that treatment with CsA appears beneficial in patients afflicted with rheumatoid arthritis or systemic lupus erythematosus [63], [64]. Although the mode of action in these studies was not investigated in detail, they may be partially attributed to the action of CsA on neutrophils in that it was observed that CSA reduced ROS production, reduced IL-8 production, chemotactic mobility and degranulation in isolated neutrophils, without altering agonist stimulated calcium liberation [41], [42], [59].

Furthermore in the treatment of steroid resistant-ulcerative colitis with CsA, it was postulated that the therapeutic benefit may in part result from reduced capacity of neutrophils to generate ROS, undergo chemotaxis or produce pro-inflammatory cytokines (IL-8) following exposure to CsA [65].

These results, therefore, suggest that it may be useful to explore the use of CsA or analogous immunosuppressive agents to modulate inappropriate or potentially deleterious NETotic events. They also provide new insight into a possible mechanism whereby patients treated with these drugs are rendered highly susceptible to opportunistic fungal infections [43].

In a similar manner, the observation that PTX abrogates NETosis may shed new light on the recent findings that infections with *Bordetella pertussis* facilitate secondary infections by suppressing neutrophil action and are cleared by reactive oxygen species [57], [58].

## Supporting Information

Video S1 **Detection of IL-8 induced NETosis by time-lapse fluorescent microscopy.** NETosis was induced with rIL-8 (100 ng/ml), and NETs were detected by fluorescent microscopy using SYTOX Green over a period of 82 minutes.
(ZIP)

Video S2 **Detection of PMA induced NETosis by time-lapse fluorescent microscopy.** NETosis was induced with PMA (50 nM) and NETs were detected by fluorescent microscopy using SYTOX Green over a period of 59 minutes.
(ZIP)
